# Eight-month functional and scar outcomes following conservative management of an extensive diabetic foot soft-tissue defect using high-purity type I collagen matrix and negative pressure wound therapy: a case report

**DOI:** 10.1093/jscr/rjag867

**Published:** 2026-09-30

**Authors:** Naveen Narayan

**Affiliations:** Department of Plastic, Reconstructive and Aesthetic Surgery, Adichunchanagiri Institute of Medical Sciences, B.G. Nagara, Nagamangala Taluk, Mandya District, Karnataka - 571448, India

**Keywords:** diabetic foot ulcer, full-thickness wound, negative-pressure wound therapy, high-purity type I collagen, Helicoll®, skin substitute, neo-epithelialization, limb salvage

## Abstract

Extensive diabetic foot defects with exposed tendons are typically managed with debridement followed by skin grafting or flap reconstruction. We report the 8-month outcome of a 45-year-old man with poorly controlled type 2 diabetes mellitus who developed a 17 × 10 cm post-infective dorsal foot defect with exposed extensor tendons after drainage of a diabetic foot abscess. The patient declined reconstructive surgery because of financial constraints and inability to interrupt daily-wage employment. Following shared decision-making, sequential applications of a high-purity type I collagen (HPTC) matrix combined with portable negative-pressure wound therapy (NPWT) achieved progressive granulation, epithelialization, and complete wound closure within 7 weeks without grafting or flap reconstruction. At 8 months, the wound remained completely healed with satisfactory scar maturation, preserved foot function, unrestricted ambulation, return to work, and no recurrent ulceration or infection. This case demonstrates the potential of an HPTC matrix combined with NPWT as a limb-salvage strategy in carefully selected patients unsuitable for conventional reconstruction.

## Introduction

Full-thickness diabetic foot wounds with exposed tendons remain a major reconstructive challenge because they are associated with prolonged healing, recurrent infection, impaired ambulation, and increased risk of amputation [1, 2]. Standard management includes prompt debridement, infection control, vascular and metabolic optimization, negative-pressure wound therapy (NPWT), and definitive reconstruction with split-thickness skin grafts or flap coverage [3–5]. Biological collagen matrices have emerged as bioactive scaffolds that support cellular migration, angiogenesis, extracellular matrix remodelling, and granulation tissue formation, making them useful adjuncts in the management of complex wounds [6–10]. Randomized studies have demonstrated improved healing outcomes in diabetic foot ulcers and full-thickness wounds using high-purity type I collagen (HPTC) matrices such as Helicoll® [11, 12]. We report the 8-month outcome of an extensive tendon-exposed diabetic foot defect that healed without skin grafting after treatment with an HPTC matrix combined with NPWT following the patient’s refusal of reconstructive surgery.

## Case presentation

A 45-year-old man with an 11-year history of poorly controlled type 2 diabetes mellitus and hypertension presented with fever, malaise, and a rapidly enlarging swelling over the dorsum of the left foot. Clinical examination revealed a fluctuant dorsal foot abscess with surrounding cellulitis and features of evolving sepsis. Emergency incision and drainage were performed, and broad-spectrum intravenous antibiotics together with intensive glycaemic control were initiated. Tissue culture grew methicillin-sensitive *Staphylococcus aureus*, and antibiotic therapy was adjusted according to culture sensitivity. Despite infection control, progressive post-infective skin necrosis resulted in an extensive full-thickness dorsal foot soft-tissue defect measuring ~17 × 10 cm with exposed extensor tendons (Fig. 1). Peripheral vascular assessment, including palpable pedal pulses, normal ankle-brachial indices, and duplex arterial Doppler, confirmed adequate limb perfusion for wound healing (Table 1).

**Figure 1:**
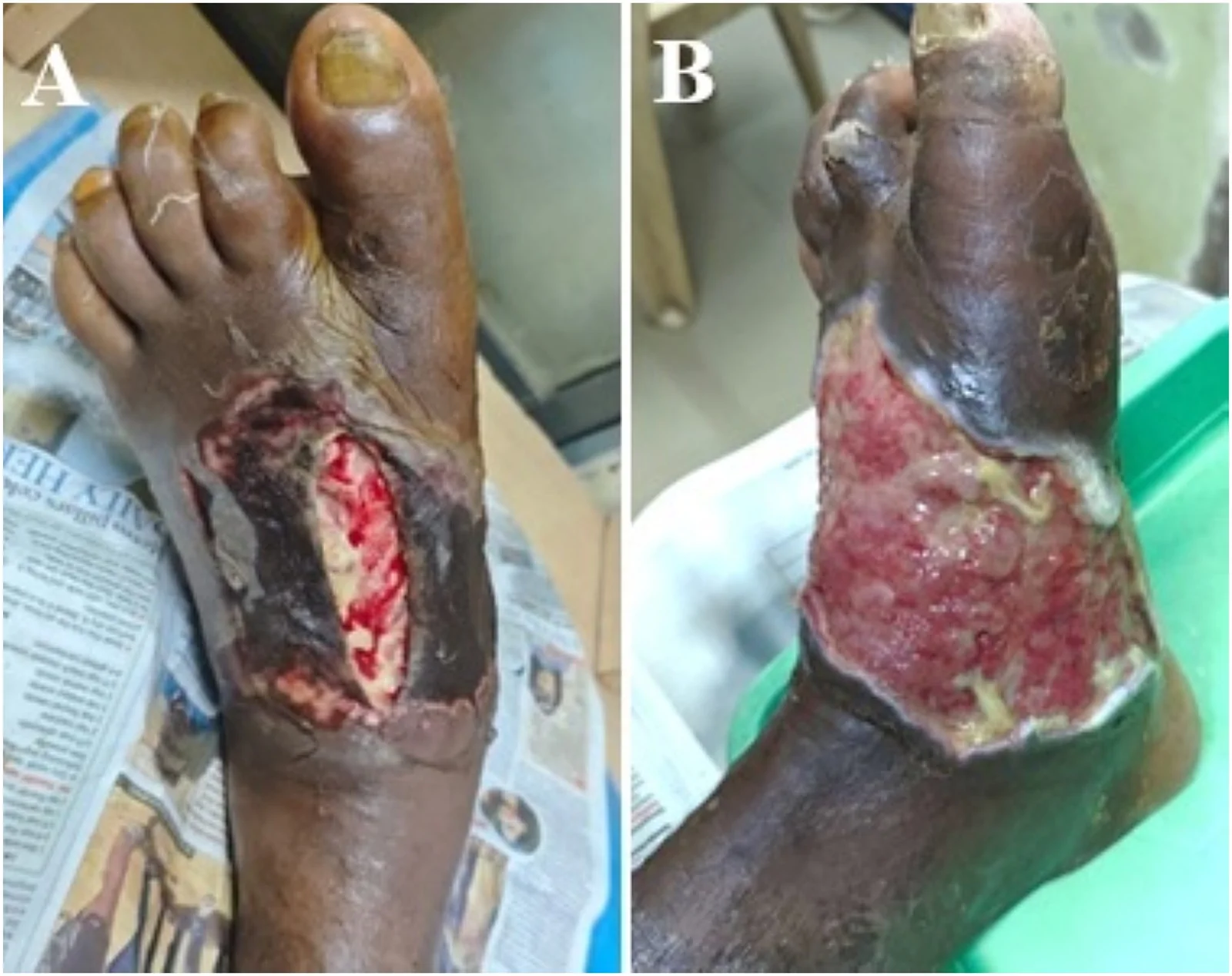
Extensive post-infective soft tissue loss following a diabetic foot abscess; (A) necrosed dorsal skin over the left foot following incision and drainage of the abscess, showing eschar; (B) after debridement and one application of HPTC with NPWT—a large (~17 × 10 cm) full-thickness raw area over the dorsum extending to the medial and volar aspects of the foot, with substantial healthy granulation tissue, coverage of exposed extensor tendons and early signs of neo-epithelialization at the margins. (Reproduced from Narayan N *et al.* under the Creative Commons Attribution 4.0 International License [11]).

**Table 1:** Baseline patient characteristics.

Variable	Value
Age	45 years
Sex	Male
BMI	27.8 kg/m^2^
Occupation	Daily-wage manual labourer
Duration of type 2 diabetes	11 years
Hypertension	Yes (6 years)
Smoking	No
Alcohol	Occasional social consumption
Peripheral neuropathy	Present (10-g monofilament sensation diminished over forefoot)
HbA1c	9.4%
Fasting blood glucose	196 mg/dl
Postprandial blood glucose	312 mg/dl
Haemoglobin	11.8 g/dl
Total leukocyte count	16 900 /mm^3^
Neutrophils	86%
CRP	126 mg/l
ESR	74 mm/h
Serum albumin	3.2 g/dl
Serum creatinine	1.0 mg/dl
eGFR	98 ml/min/1.73 m^2^
Dorsalis pedis pulse	Palpable
Posterior tibial pulse	Palpable
ABI (left)	1.02
ABI (right)	1.05
Duplex arterial Doppler	Normal triphasic flow without significant stenosis

Baseline demographic characteristics, comorbidities, metabolic profile, laboratory investigations, and vascular assessment of the patient at presentation before initiation of conservative limb-salvage treatment. BMI, body mass index; HbA1c, glycated haemoglobin; CRP, C-reactive protein; ESR, erythrocyte sedimentation rate; eGFR, estimated glomerular filtration rate; ABI, ankle-brachial index.

Standard treatment comprising serial debridement, NPWT, and definitive split-thickness skin grafting was recommended. However, after detailed counselling regarding the risks and benefits, the patient declined reconstructive surgery because of financial constraints and the inability to interrupt his daily-wage employment. Following a multidisciplinary discussion and shared decision-making, a conservative limb-salvage strategy was undertaken.

After meticulous surgical debridement and wound-bed preparation, sequential applications of an HPTC matrix (Helicoll®, Encoll Corp., Fremont, CA, USA) were combined with portable NPWT. The wound was reviewed at regular intervals, and collagen applications were repeated according to the development of healthy granulation tissue and the absence of active infection. Glycaemic control, nutritional support, off-loading, and culture-directed antimicrobial therapy were maintained throughout treatment.

Healthy granulation tissue progressively covered the exposed extensor tendons, followed by wound contraction and advancing epithelialization. Four sequential collagen applications were performed over 7 weeks, resulting in complete wound closure without the need for split-thickness skin grafting or flap reconstruction (Fig. 2). The patient gradually progressed from protected weight-bearing to unrestricted ambulation and resumed his occupational activities after complete wound healing. The chronological treatment course and clinical progress are summarized in Table 2.

**Figure 2:**
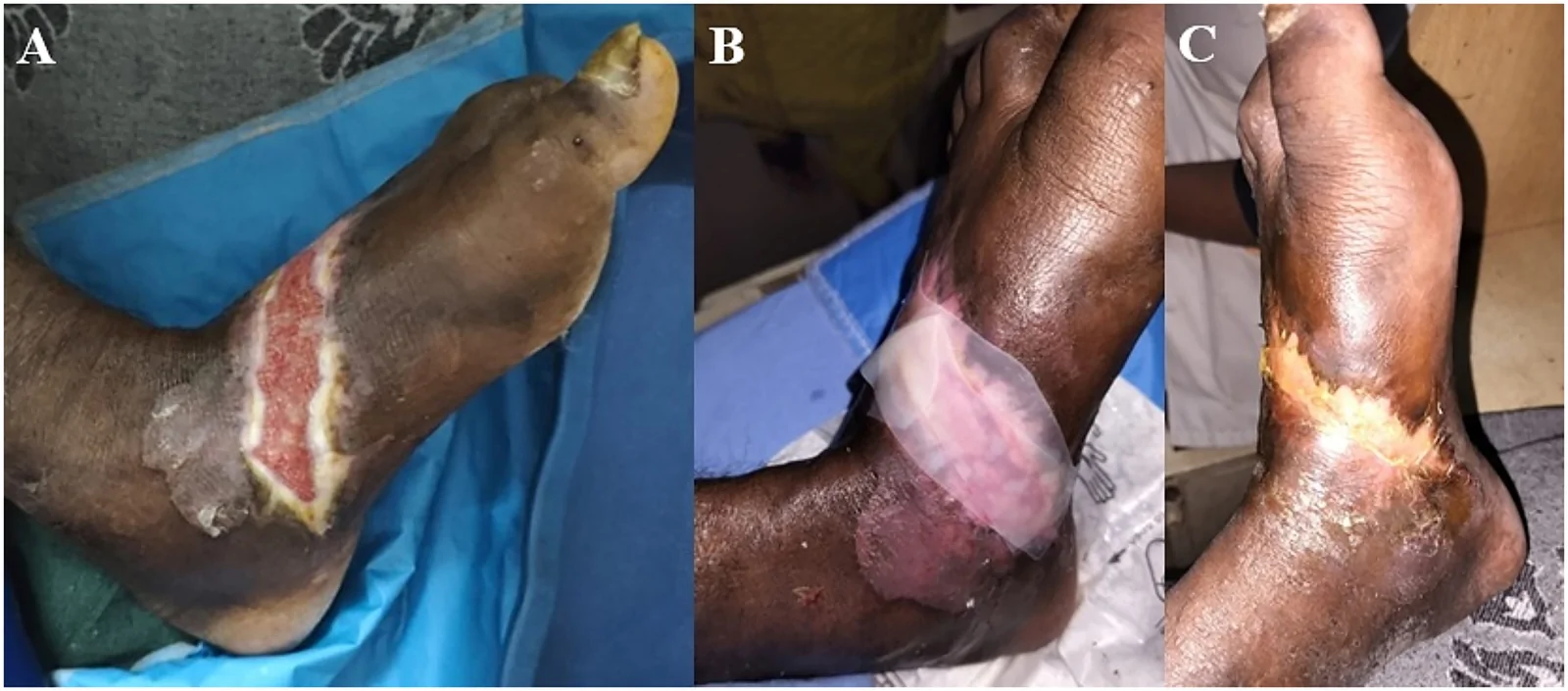
Progressive regeneration following sequential HPTC and NPWT applications; (A) wound status at 5 weeks demonstrating healthy granulation tissue formation, wound contraction (more than 60%–70% decrease in size), and peripheral epithelial advancement following three applications of HPTC with NPWT; (B) fourth application of the HPTC membrane (translucent collagen sheet) over the wound prior to the fourth NPWT cycle; (C) complete wound closure at 7 weeks, leaving a single linear scar across the dorsum. (Reproduced from Narayan N *et al.* under the Creative Commons Attribution 4.0 International License [11]).

**Table 2:** Clinical timeline.

Time	Intervention	Outcome
Admission	I&D + IV antibiotics	Sepsis controlled
Week 1	First HPTC + NPWT	Healthy granulation
Week 3	Second HPTC + NPWT	Progressive epithelialization
Week 5	Third HPTC + NPWT	>70% wound area reduction
Week 7	Fourth HPTC + NPWT	Complete closure
Month 8	Follow-up	Stable scar, normal function

I&D, incision and drainage; IV, intravenous.

At the 8-month follow-up, the wound remained completely healed with durable soft-tissue coverage, satisfactory scar maturation, a preserved foot contour, unrestricted ankle and toe movement, and no recurrent ulceration, infection, tendon exposure, or requirement for additional reconstructive procedures (Fig. 3). Long-term functional and clinical outcomes at 8 months are summarized in Table 3.

**Figure 3:**
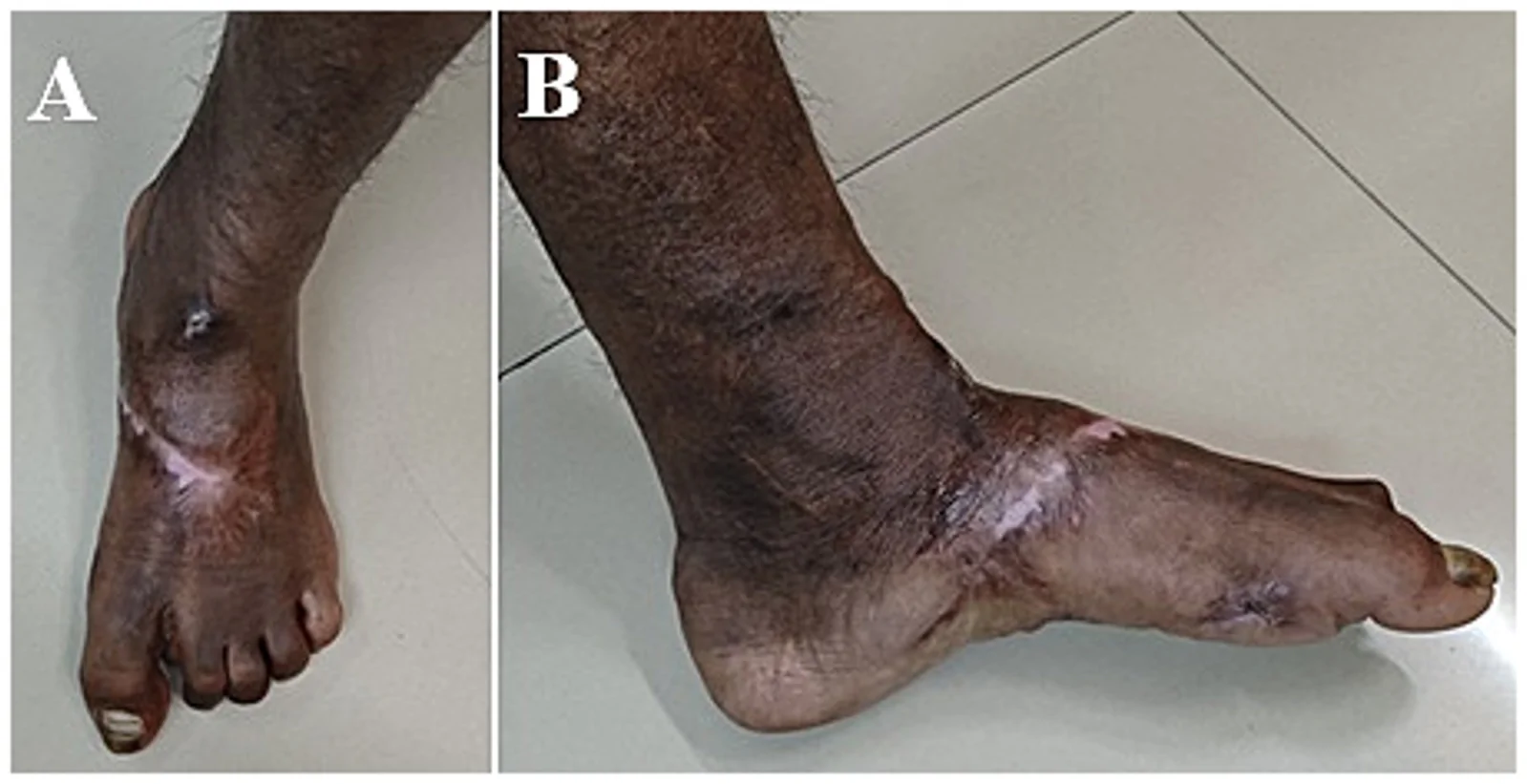
Eight-month follow-up demonstrating durable regenerative healing and scar maturation; (A) dorsal view and (B) medial/oblique view of the left foot at 8 months, showing the matured linear scar with regenerated skin of near-normal colour, tone, and pliability; there is no contracture, no hypertrophic scarring, and preserved foot contour and function—consistent with true neo-epithelialization rather than simple contraction.

**Table 3:** Eight-month outcome.

Outcome	Result
Wound closure	Complete
Scar (VSS)	2/13
Ulcer recurrence	None
Infection	None
Footwear	Regular
Ambulation	Unrestricted
Return to work	Full
Need for skin graft/flap	Not required
Additional surgery	Not required

Summary of clinical, functional, and scar outcomes at 8-month follow-up after conservative limb-salvage treatment. VSS, Vancouver Scar Scale.

The Vancouver Scar Scale score was 2/13, indicating an excellent scar outcome, and the patient was able to wear regular footwear and return to full-time work without functional limitation (Table 4).

**Table 4:** Vancouver Scar Scale assessment of the regenerated dorsal scar at 8-month follow-up (lower scores indicate a better scar; maximum 13).

Parameter	Score	Clinical interpretation	Maximum (worst) score
Vascularity	0	Normal vascularity	3
Pigmentation	1	Mild residual dyschromia	2
Pliability	1	Supple scar	5
Height	0	Flat scar	3
**Total VSS Score**	**2/13**	**Excellent scar outcome**	13

## Discussion

Extensive diabetic foot wounds with exposed tendons are conventionally managed using repeated debridement, infection control, vascular and metabolic optimization, followed by definitive soft-tissue reconstruction with split-thickness skin grafts or flap coverage when primary closure is not feasible [3–5, 12, 13]. However, reconstructive surgery may not be possible in patients with significant comorbidities, financial limitations, or refusal of further operative intervention.

In this patient, adequate vascular perfusion and successful infection control provided a favourable environment for conservative limb salvage. Sequential application of an HPTC matrix combined with NPWT resulted in progressive granulation tissue formation, epithelialization, and durable wound closure without skin grafting or flap reconstruction. Collagen matrices provide a temporary extracellular matrix scaffold that supports cellular migration, angiogenesis, and organized tissue repair, while NPWT promotes wound-bed preparation and granulation tissue formation [3–11, 14–19]. The favourable 8-month outcome, including stable wound closure, satisfactory scar maturation, preserved foot function, and a return to unrestricted ambulation, suggests that this combination may be a useful adjunct in carefully selected patients who decline standard reconstructive procedures.

Although our previous randomized clinical trials demonstrated improved short-term healing with the same collagen matrix in diabetic foot ulcers and full-thickness wounds [8, 9, 11, 16], this report highlights sustained long-term functional recovery and scar quality in a single patient with an unusually extensive tendon-exposed defect.

The sustained 8-month follow-up demonstrating durable wound closure, absence of recurrent ulceration, preserved tendon function, unrestricted ambulation, and return to regular employment extends the evidence beyond short-term wound healing and highlights the potential durability of this treatment strategy in selected patients.

To our knowledge, this represents one of the few reports describing the durable 8-month healing of a large tendon-exposed diabetic foot defect managed conservatively with a sequential HPTC matrix and NPWT without skin grafting or flap reconstruction.

This case should be interpreted within the limitations of a single observation and does not establish comparative efficacy or causality. Larger prospective studies with objective functional, histological, and patient-reported outcome measures are required to define the role of collagen-based biological scaffolds as adjuncts in complex diabetic foot reconstruction [18–20].

## Conclusion

This case demonstrates that sequential application of an HPTC matrix combined with NPWT achieved durable wound healing, satisfactory scar maturation, and preserved function in an extensive tendon-exposed diabetic foot defect after the patient declined reconstructive surgery. This approach may be considered as an adjunctive limb-salvage option in carefully selected patients, although larger prospective studies are needed to confirm its effectiveness.

## Data Availability

All data generated or analysed during this study are included in this published article. Additional de-identified information is available from the corresponding author upon reasonable request.
